# Effect of Adjuvant on Glyphosate Effectiveness, Retention, Absorption and Translocation in *Lolium rigidum* and *Conyza canadensis*

**DOI:** 10.3390/plants9030297

**Published:** 2020-03-01

**Authors:** Candelario Palma-Bautista, Jose G. Vazquez-Garcia, Ilias Travlos, Alexandros Tataridas, Panagiotis Kanatas, José A. Domínguez-Valenzuela, Rafael De Prado

**Affiliations:** 1 Department of Agricultural Chemistry and Edaphology, University of Cordoba, 14071 Cordoba, Spain; z82pabac@uco.es (C.P.-B.); z82vagaj@uco.es (J.G.V.-G.); 2 Department of Crop Science, Laboratory of Agronomy, Agricultural University of Athens, 75 Iera Odos Street, 11855 Athens, Greece; travlos@aua.gr (I.T.); a.tataridas@gmail.com (A.T.); 3 Agricultural Cooperative of Mesolonghi-Nafpaktia, 30200 Mesolonghi, Greece; pakanatas@gmail.com; 4 Department of Agricultural Parasitology, Chapingo Autonomous University, 56230 Texcoco, Mexico; jose_dv001@yahoo.com.mx

**Keywords:** rigid ryegrass, horseweed, efficacy, retention, absorption and translocation

## Abstract

Glyphosate retention, absorption and translocation with and without adjuvant were examined in *Lolium rigidum* and *Conyza canadensis* in greenhouse and laboratory settings to develop an understanding of the influence of the selected adjuvant on glyphosate activity. Tests on whole plants show that the dose of herbicide needed to reduce dry weight by 50% (GR_50_) or plant survival (LD_50_) decreases by mixing glyphosate and adjuvant to 22%–24% and 42%–44% for both populations of *L. rigidum* and *C. canadensis*, respectively. This improvement in efficacy could be attributed to the higher herbicide retention and lower contact angle of the glyphosate + adjuvant drops on the leaf surface compared to the glyphosate solution alone. Plants of both species treated with ^14^C-glyphosate + adjuvant absorbed more glyphosate compared to non-adjuvant addition. Furthermore, the movement of the herbicide through the plant was faster and greater with the adjuvant. Our results reveal that the use of adjuvants improves the effectiveness of glyphosate in two of the most important weeds in agricultural crops in Mediterranean countries.

## 1. Introduction

Glyphosate [N-(phosphonomethyl) glycine] has been used worldwide for several decades in a wide range of agricultural and non-agricultural situations, with glyphosate-based herbicides serving as broad-spectrum, water-soluble, non-selective, systemic, post-emergence herbicides [1,2,3,4]. Its mode of action is the inhibition of the shikimic acid pathway, blocking the synthesis of the aromatic amino acids (AAA) phenylalanine (Phe), tryptophan (Trp), and tyrosine (Tyr) [2,3].

Glyphosate is considered as the most important herbicide globally [2], which has led to overreliance on it. Repeated application of glyphosate has contributed to the widespread occurrence of glyphosate resistance in several weed species, with 48 glyphosate-resistant (GR) weed species reported [5]. Currently, there are more than five hundred cases worldwide of weeds which have evolved herbicide resistance [5].

A proactive approach to preventing GR weeds could be achieved by including alternative herbicides with different mechanisms of action, by improving herbicide efficacy and through the introduction of Integrated Weed Management (IWM) strategies. However, glyphosate still remains a very effective tool, especially for perennial weeds, and considerable research has been conducted regarding the enhancement of glyphosate-based herbicide formulations [2]. Tank mixtures and formulated products including adjuvant additives act in a way that is beneficial for glyphosate applications [2]. In general, one key element for the improvement of herbicide efficacy is the increase in absorption into plant tissues [6]. The addition of adjuvants to glyphosate may increase herbicide uptake by acting on the leaf surface, on the cuticle and within the internal tissues [2,3]. However, it is noted that the synergistic effects of glyphosate and adjuvants are strongly influenced and depend on several factors, including the nature of the leaf surface, the relative humidity and the physicochemical properties of the formulated product and adjuvants [3,7].

Adjuvants are additives either in herbicide formulations or in spray tank mixes, which interact with plant tissues in various ways [8,9]. Their main function is to improve herbicide efficacy, a trait that is carried out through modifications in herbicide properties via spray droplet formation and impaction, coverage and deposit formation, as well as wetting performance and spray retention of the leaf surface [2,3,10].

Surfactants are a common category of adjuvants. They increase herbicide active ingredient utilization by enhancing droplet deposition, spread and retention on all leaf surfaces, leading to an increase in herbicide uptake by the plants [11]. More specifically, surfactants are added to pesticide solutions in order to lower the surface tension of the spray solution and thereby increase surface wetting [2,9,11]. Some of them may act as penetrants, enhancing the solubility of the herbicide in the cuticular waxes and affecting epicuticular wax morphology [2,9]. Nevertheless, attention must be paid to the selection of the surfactant and the determination of its concentration to increase the leaf retention and translocation of pesticides in order that application rates and production costs remain low [9,11].

Surfactants are categorized as cationic, anionic, non-ionic and amphoteric, with the most commonly used being the non-ionic group [1,11]. Non-ionic surfactants have been reported to minimize droplet rebound, increase droplet spreading, enhance retention and improve the performance of active ingredients in spray mixtures [1,12].

*Lolium rigidum* Gaud. (rigid ryegrass) belongs to the Poaceae family and is considered to be a noxious winter annual weed in the Mediterranean basin [13,14]. The most common practices that are implemented against rigid ryegrass populations include chemical applications with glyphosate and, in addition, acetyl-CoA carboxylase (ACCase) and acetolactate synthase (ALS) inhibitors [14]. *L. rigidum* presents high fecundity, genetic variability and phenotypic plasticity, as well as prolific seed production—traits which give rigid ryegrass the ability to evolve multiple resistances to herbicides and distribute broadly [14,15]. Currently, 48 unique cases of herbicide-resistant rigid ryegrass have been reported worldwide, while glyphosate resistant populations seem to be widespread in Spain [5,13].

*Conyza canadensis* (L.) Cronquist (horseweed) is mainly an annual herbaceous weed of North American origin, present in many regions in the Mediterranean zone. It also has high seed production, with up to 200,000 seeds per individual plant, while seed dispersal through wind has been measured to cover a distance of 500 m from the source plant [16,17,18] and is able to germinate in a broad spectrum of environmental conditions [19]. *C. canadensis* is one of the most reported weed species for herbicide resistance, while populations of this species in orchards have evolved resistance to EPSP (5-enol-pyruvyl-shikimate-3-phosphate) synthase inhibitors in Spain, Greece and Italy [5].

Across the Mediterranean countries, the main weed management option still remains herbicide application. Often, farmers aim to manage noxious weeds, such as rigid ryegrass and horseweed, in late growth stages, reducing the potential efficacy of the applied herbicides and resulting in overreliance on glyphosate. Glyphosate is a tool that is broadly used by farmers in order to control horseweed and rigid ryegrass, especially in low tillage systems such as orchards. The efficacy of glyphosate in weed species is strongly associated with the growth stages of the species [16,17]. There is a need to maintain the efficacy of glyphosate in desirable levels without increasing the applied doses. This demand may be addressed with a right choice and use of an adjuvant.

The scope of this study was to evaluate the effectiveness of an adjuvant, when this is added to glyphosate formulations, relating to glyphosate retention, absorption and translocation in *L. rigidum* and *C. canadensis* populations. The objectives of this study were as follows: (1) to determine the susceptibility of *L. rigidum* and *C. canadensis* populations in the three to four leaf stage and rosette stage, respectively, to glyphosate in greenhouse conditions and evaluate the efficacy of an adjuvant when this is added to the formulation; and (2) to characterize physical (foliar retention and contact angle) and physiological (absorption and translocation of [^14^C] glyphosate) factors that could explain the differential sensitivity to glyphosate plus the adjuvant in rigid ryegrass and horseweed.

## 2. Results

### 2.1. Dose–Response Assay

**Figure 1:**
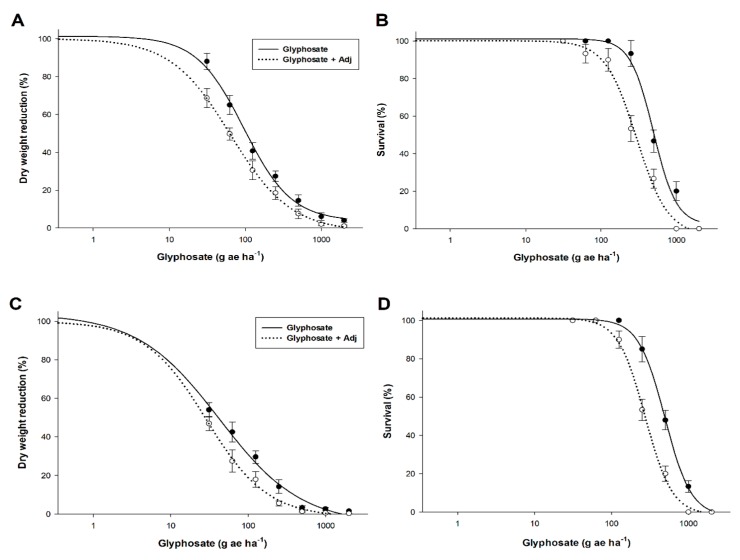
Glyphosate dose response in dry weight reduction and survival expressed as a percentage of the mean untreated control of *C. canadensis* (**A** and **B**) and *L. rigidum* (**C** and **D**).

Under greenhouse conditions, *C. canadensis* and *L. rigidum* exhibited a high sensitivity to glyphosate at the rosette and 3–4 leaf growth stage, respectively, showing very low LD_50_ values compared to 1080 g ae (acid equivalent) ha^−1^—the field dose generally used by farmers in agricultural crops (Table 1 and Figure 1B,D). Sensitivity differences (GR_50_) were apparent among species, and the dicot *C. canadensis* was 2.6 times less susceptible to glyphosate than the grass weed *L. rigidum*. The effectiveness of glyphosate clearly increases with the addition of an adjuvant (Adj) in both species (Table 1 and Figure 1A,C). The increase in the effectiveness of Adj [(GR_50_ gly-GR_50_ gly + adj) / (GR_50_ gly)]*100 or [(LD_50_ gly-LD_50_ gly + adj) / (LD_50_ gly)]*100 in both weed species was similar. However, the effectiveness was almost double considering LD_50_ values in the case of GR_50_ for both species.

### 2.2. Spray Retention Assays

Spray retention and contact angle are important parameters of herbicide efficacy, because they determine the maximum amount of herbicide that can penetrate the plant surface [20,21]. In both *C. canadensis* and *L. rigidum* populations, glyphosate retention and the leaf contact-angle were inverse when comparing plants treated with glyphosate alone or glyphosate plus adjuvant (Table 2). These results explain that the higher the amount of retained glyphosate, the lower the contact angle droplets exhibit on the leaf surface, and they can expand, achieving a greater foliar contact surface.

### 2.3. ^14^C-Glyphosate Absorption and Translocation

**Figure 2:**
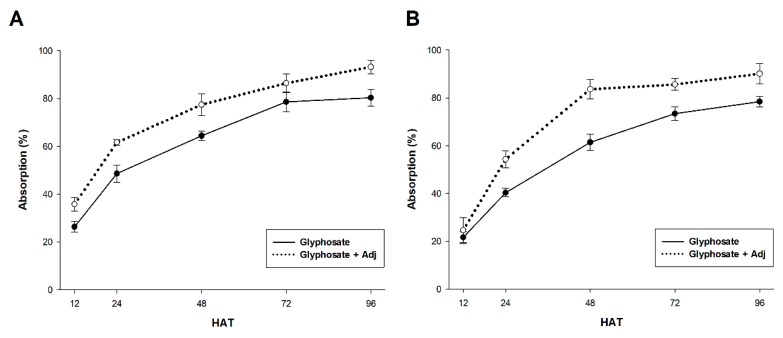
Absorption of ^14^C-glyphosate from 12 to 96 h after treatment (HAT) in *C. canadensis* (**A**) and *L. rigidum* (**B**) populations. The vertical bars represent the standard error of the mean (n = 5).

**Figure 3:**
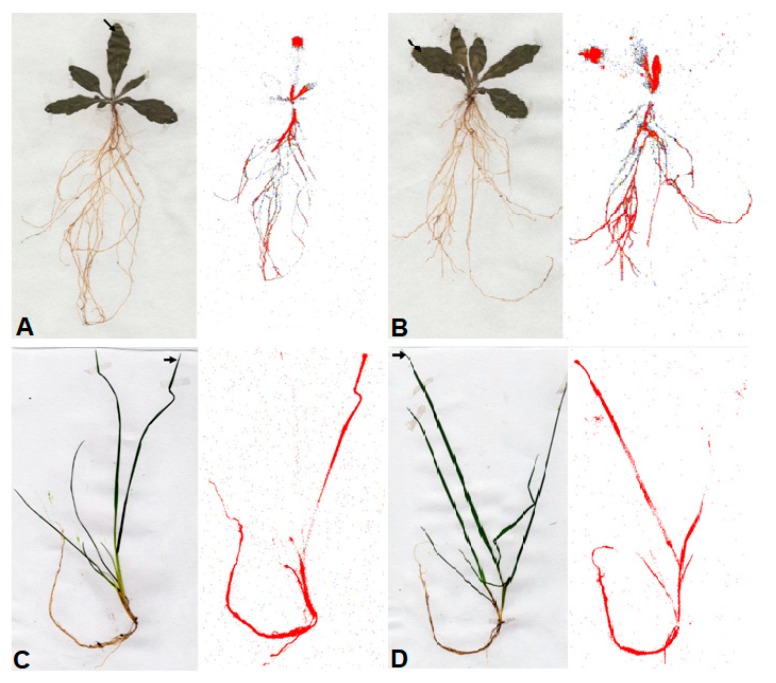
Digital image (plants on the left) and autoradiography (plants on the right) show the distribution of ^14^C-glyphosate (**A** and **C**) and ^14^C-glyphosate + Adj (**B** and **D**) within *C. canadensis* and *L. rigidum* populations, at 96 HAT.

There were significant differences in the leaf absorption of applied ^14^C-glyphosate and ^14^C-glyphosate + Adj in *C. canadensis* and *L. rigidum* for all evaluation timings (Figure 2A,B). Clearly, these results, together with those found for herbicide retention and contact angle, could explain the best control of these weed species using the mixture of glyphosate plus adjuvant. In addition, the translocation of glyphosate 96 h after treatment (HAT) from the treated leaf (TL) to the rest of the plant (RP+RS) is also improved with the addition of the adjuvant to the glyphosate solution in both species analyzed (Table 3). The differences in translocation of ^14^C-glyphosate and ^14^C-glyphosate + Adj in the *C. canadensi*s and *L. rigidum* plants were also visualized by phosphor images (Figure 3). In general, the translocation of ^14^C-glyphosate 96 HAT to roots was different, with lesser glyphosate accumulation compared to the mixture with the adjuvant being clearer in *C. canadensis* than for *L. rigidum* plants (Figure 3B,D). This visual difference was consistent with the differences observed in glyphosate retention, absorption and translocation.

## 3. Discussion

Globally, extreme efforts from farmers, companies and agricultural organizations have been made to enhance the efficacy of herbicides. One key factor that leads to improved herbicide application is the improvement of leaf absorption of herbicides. The absorption process requires thorough research around the various parameters that affect retention, spreading, penetration, and finally the translocation of pesticide substances within the plant tissues. Therefore, some of the important parameters that should be taken into account are the specific characteristics of the plant surface (i.e., wax composition and leaf roughness) and the traits of the sprayed liquid [6]. Regarding the leaf surface characteristics that affect spray adhesion, the trichomes, the cuticular membrane and the extent of wax act as interceptors to droplet spreading [22].

The need for the addition of adjuvants to glyphosate solutions was previously reported by Castro et al. [1], who claimed that glyphosate alone or in its different salt forms could not effectively penetrate specific structural elements of plant surfaces. This was also supported by Li et al. [6], who reported that an added surfactant leads to improved droplet coverage on leaves and therefore to better absorption of glyphosate.

Plant species present different wettability in their leaves, due to a different leaf architecture such as leaf roughness and wax [23]. When roughness is high, the wettability remains low [24]. Under this concept, plant species’ surfaces can be classified from easy-to-wet through to difficult-to-wet, describing why some plants show very non-wetting properties and lead to a low retention of the sprayed liquids. [25,26]. 

Droplet contact angles which range between 600 and 800 could be characterized as moderate wetting [27,28]. In this research, contact angle measurements showed that, for *C. canadensis* and *L. rigidum*, the ranges were between 650 and 740 and 680 and 740, respectively. It is obvious that both species surveyed showed a low droplet contact angle—a trait that indicates that these species have moderately easy-to-wet leaf surfaces. The addition of the adjuvant led to desirable results, because it reduced the contact angle in both species, which led to the better spreading of the droplet and an increased wetted area [11,29,30]. Menendez et al. [30] also categorized *L. rigidum* as a moderately rough-surfaced grass weed, and with the addition of adjuvants was able to lower the glyphosate contact angle values by up to 23% in comparison to the control population, while the leaf coverage and glyphosate uptake was enhanced. The use of adjuvants could decrease the contact angle even in superhydrophobic leaf surfaces, such as rice leaves, increasing the wettability severely [31].

Our results indicate that 96 HAT glyphosate uptake is increased—a result that is similar to Fernandez Moreno et al. [13], who tested the uptake and translocation of glyphosate in a susceptible and a resistant population of rigid ryegrass in Spain. A possible justification as to why the adjuvant increased the glyphosate uptake by the plants is that the capacity of surfactants with wetting properties to maintain the moistened droplets increased, extending the time of retention and uptake by the leaf surfaces [1]. Menendez et al. [30] concluded that contact angle is the most important factor affecting the retention of glyphosate and its efficacy in a moderate rough surfaced of *L. rigidum.*

The dose–response assay revealed that the dicot horseweed was less susceptible to glyphosate than rigid ryegrass. This can be explained by the differences in the outer surfaces of leaves of these two weeds and the different leaf plant structures. In both species, however, the addition of the adjuvant increased the sensitivity to glyphosate. Regarding the survival of the surveyed species, the rigid ryegrass population tested is confirmed to be less susceptible to glyphosate, presenting a LD_50_ value of 469 g ae ha^−1^, in comparison with the results of Fernandez Moreno et al. [13], where a susceptible population had a LD_50_ value of 118 g ae ha^−1^. Our results show that the achieved LD_50_ values in horseweed and rigid ryegrass are significantly lower than the common field dose of glyphosate (1080 g ae ha^−1^), when glyphosate is mixed with an adjuvant.

The absorption of ^14^C-glyphosate was relatively high for both species, with an increasing tendency, even at 96 HAT. The addition of the adjuvant improved the absorption rate and led to the better translocation of glyphosate to the roots. These results can be supported by the outcomes of Gonzalez-Torralva et al. [16] regarding absorbed and translocated glyphosate as a sole application in a susceptible population of *C. canadensis,* which showed low susceptibility to glyphosate with no significant translocation to roots. Concerning the translocated glyphosate in rigid ryegrass, Fernandez Moreno et al. [13] revealed that at 96 HAT there was an increase in translocation from the treated leaf to the rest of the plant tissues and the roots in a glyphosate-susceptible rigid ryegrass population—a pattern similar with our results when the adjuvant was added to the solution. 

Our results demonstrate that the adjuvant interacted with the leaves of both rigid ryegrass and horseweed and improved the efficacy of glyphosate. The added adjuvant INEX-A increased the retention, lowered the contact angle in the leaf surfaces, improved the absorption of glyphosate and led to the better translocation of glyphosate from the leaves to the whole plant. The outcomes of this research imply that the utilization of non-ionic surfactants could increase the efficacy of glyphosate on several weed species. This result is supported by the fact that a dose of 280 g ae ha^−1^ glyphosate leads to a 50% survival reduction in *C. canadensis* and *L. rigidum.* This result compares to the common field rate of glyphosate applied against annual and perennial weeds by farmers in agricultural crops in Spain. The addition of adjuvants in spray solutions could pose a key factor, meaning that herbicide costs can be reduced and a proper control of certain noxious weeds can be achieved without increasing the applied doses of herbicides. This study highlights that, for a better use of glyphosate, the addition of an adjuvant could be beneficial, not only for the herbicide efficacy against weeds, but also for the environment due to lower applied doses of herbicide. Nevertheless, further research should be conducted on the effects of glyphosate formulations with the addition of surfactants to ecosystems, because these substances could be environmentally detrimental [32].

Regardless of the vulnerabilities of glyphosate which have been reported in recent decades, this herbicide still remains a very effective tool against many annual and perennial weeds; its utilization, however, should be further investigated [1,2]. The use of adjuvants with herbicide active ingredients has been long reviewed and reported. Future research must include an analysis of the interactions between herbicides, adjuvants and specific plant species [1,33], so that we may deeply understand the complex positive—or, in some cases, detrimental—effects resulting from the utilization of surfactants

## 4. Materials and Methods 

### 4.1. Chemical

^14^C-glyphosate (specific activity 273.8 MBq mmol−1, 95% radio-chemical purity) was obtained from American Radiolabeled Chemicals, Inc. (Saint Louis, MO, USA). A commercial formulation (Roundup Energy 45% (w/v) SL) of the herbicide was supplied by Monsanto Agricultura España S. L. and used in all experiments described below. The adjuvant (Adj: INEX-A, Cosmocel. Mexico) was a mixture of the ethoxyl alcohols poliglicol and aril polyethoxyethanol of a non-ionic nature with the power of humidification (penetration) and dispersion, which serves as an adjuvant in the application of agrochemicals in general. It is a product designed to ensure and/or improve the effectiveness of sprayed agrochemicals where greater humidification and coverage is required.

### 4.2. Plant Material

Seeds of 25 plants of *L. rigidum* and *C. canadensis* were harvested from an organic olive orchard which had never received herbicide application during the past 20 years.

Seeds from the susceptible populations were sown in pots that were filled with moistened peat and covered with a transparent film up to emergence (2 cm). The seedlings were individually transplanted into 7 × 7 × 7 cm pots with a 1:1 (v/v) mixture of peat–sandy soil and placed in a greenhouse at 26/18 °C (day/night), with a 16 h photoperiod, 850 μmol m^−2^ s^−1^ photosynthetic photon flux density, and 80% relative humidity.

### 4.3. Dose–Response Assay

Herbicide treatments were applied at the 3–4 leaf growth stage. Glyphosate formulations with or without adjuvant (4mL L^−1^) were applied within a laboratory chamber (SBS-060 De Vries Manufactering, Hollandale, MN) equipped with 8002 flat fan nozzles delivering 200 L ha^−1^ at 250 kPa at the height of 50 cm. The following glyphosates rates were used: 0, 31.25, 62.5, 125, 250, 500, 1000, 2000 g ae ha^−1^; the experiment was arranged in a completely randomized design using five replicates per rate. The dry weight of plants was measured for aboveground parts of *L. rigidum* and *C. canadensis* plants and evaluated 21 days after application (DAA). The assay was repeated twice at different times.

### 4.4. Spray Retention and Contact Angle Assays

The methodology described by Amaro-Blanco et al. [34] was followed. Rigid ryegrass and horseweed plants at the 3–4 leaf and rosette stage (BBCH 16-18) [35], respectively, were sprayed with glyphosate formulation with or without adjuvant (4mL L^−1^) using the spray chamber as described above. Treatment solutions contained glyphosate formulations at 360 g ae ha^−1^ in a volume of 200 L and 100 mg L^−1^ Na-fluorescein. After two hours, the solution had dried on the foliage, and plants were cut off at ground level and immersed for 30 s in 50 mL of 5 mM NaOH. Readings were made with a spectrofluorimeter at 490/510 nm. Plants were then placed at 80 °C for 48 h, and the dry matter was recorded. Four replications were used for each treatment and the experiment was repeated twice at different time.

The third leaf of each species described above was cut off and placed onto a horizontal surface. Each leaf was treated with one 1 μL droplet containing glyphosate corresponding to 360 g ae ha^−1^ in a volume of 200 L. Droplets were applied in the center of the adaxial surface [16]. The pattern of droplet deposition was observed in a horizontal microscope (LeicaMZ6 1,8X-4X). Images were captured with a camera [LeicaDigilux 4.3 (1:2-8-4.5/8.3-24.9mm) + Supermacro Leica Digimacro 4.3] adapted to one of the oculars of the microscope. The droplets were applied every 18 s. The contact angle was obtained by digital image analysis using the ImageJ program [16]. Twenty-five replicates were measured for each *L. rigidum* and *C. canadensis* populations.

### 4.5. ^14^C-Glyfosate Absorption and Translocation

The methodology described by Fernandez-Moreno et al. [13] and Amaro-Blanco et al. [34] with some modifications was followed. The glyphosate formulation with or without adjuvant (4mL L**^−^**^1^) at the rate of 360 g ae ha^−1^ in a volume of 200 L was mixed with radiolabel ^14^C-glyphosate in order to prepare a solution with an activity of 0.834 kBq µL^−1^. Both glyphosate-susceptible plants were treated with the radiolabeled solution when they reached the 4–6 leaf stage by applying one droplet of 1.0 µL in the third leaf of each plant. The plants were kept outside the growth chamber as long as the droplet had dried on the application site; later, the plants were taken back. At different time intervals of droplet application (24, 48, 72 and 96 h), the treated leaf was washed in batches with 3 mL of a water–acetone (9:1 v/v) solution in order to quantify the unabsorbed ^14^C-glyphosate. Then, 7 mL of scintillation liquid was added to each rinse, and the radioactivity was measured by liquid scintillation spectrometry using a Scintillation Counter LS 6500 (Beckman Coulter) instrument. Similarly, ^14^C-glyphosate translocation in plant 96 HAA was divided into the treated leaf, the rest of the shoot and roots, and placed into cellulose cones. After this, cellulose cones with the different fresh tissues were dried at 60 °C for 72 h, and were combusted in a biological sample oxidizer (307, PerkinElmer). The ^14^CO_2_ was trapped and mixed with 18 mL of a 9:9 v/v mixture of Carbo-Sorb^®^ E and Permaf1uor^®^ (PerkinElmer), then quantified by liquid scintillation spectrometry as stated before. The experiment was arranged in a completely randomized design using three replicates per population and the experiment was repeated twice.

In addition, the ^14^C-glyphosate translocation was visualized using a Phosphor Imaging (Cyclone, Perkin Elmer, Packard Bioscience BV). After the mentioned period (96 HAA) had passed, whole plants were washed, fixed on filter paper (25 x 12.5 cm), dried at room temperature for 4 days, and placed on a film with phosphor crystals (AGFA CURIX) for 4 h. Three plants were used for each population and the experiment was repeated twice.

### 4.6. Data Analysis

Data percentages for dry weight reduction and survival were submitted to a non-linear regression analysis to find out the amount of glyphosate needed to reduce the dry weight (GR_50_) and cause mortality (LD_50_) of each rigid ryegrass and horseweed populations. The three-parameter log-logistic equation (1) was used:

Y = [(d)/1 +(x/g)^b^], (1)

where Y is the percentage of dry weight and mortality relative to the control; x is the herbicide rate; d is the upper limit; g is the GR_50_ or LD_50_; and b is the curve slope in g. Analyses were conducted using the drc package [36] with program R, version 3.6.0, and the data were plotted using SigmaPlot 12.0 (Systat Software, Inc.).

Data of glyphosate retention, ^14^C-glyphosate absorption and translocation were subjected to analysis of variance (ANOVA) using Statistix, version 10.0, from Analytical Software (Tallahassee, FL). Model assumptions of normal distribution of errors and homogeneous variance were graphically inspected. Differences with *p* < 0.05 were considered significant and Tukey’s test was conducted for means comparison.

## 5. Conclusions

Major efforts from farmers, companies and agricultural organizations refer to the enhancement of herbicide efficacy. One key aspect to improving herbicide performance is the increase in the absorption and translocation of herbicides to target-site plants. Our results reveal that the use of adjuvants (INEX-A) increases the effectiveness of glyphosate on *C. canadensis* and *L. rigidum*—some of the most important weeds in agricultural crops in Mediterranean countries.

## Figures and Tables

**Table 1:** Parameters of the log-logistic equations^a^ used to calculate the glyphosate rates (g ae ha^−1^) required for 50% reduction in dry weight (GR_50_) or survival (LD_50_) of *C. canadensis* and *L. rigidum* populations.

**GR_50_**						
Species	Treatment	b	d	GR_50_ *(g ae ha^−1^)*	*P*-value	IE
*C. canadensis*	Glyphosate	1.56	100.88	95.86 ± 5.98	<0.001	-
	Glyphosate + Adj	0.96	98.99	72.64 ± 6.99	<0.001	24.22
*L. rigidum*	Glyphosate	0.99	100.99	36.56 ± 3.99	<0.001	-
	Glyphosate + Adj	1.03	98.86	28.18 ± 3.32	<0.001	22.92
**LD_50_**						
Species	Treatment	b	d	LD_50_ *(g ae ha^−1^)*	*P*-value	IE
*C. canadensis*	Glyphosate	2.81	96.38	493.89±16.52	<0.001	-
	Glyphosate + Adj	2.38	101.68	283.06±18.22	<0.001	42.52
*L. rigidum*	Glyphosate	3.45	100.56	468.98±23.87	<0.001	-
	Glyphosate + Adj	2.69	100.99	275.88±17.06	<0.001	41.17

^a^ Y = [(d)/1 +(x/g)^b^] where: d is the coefficient corresponding to the upper asymptote, b is the slope of the line, x the herbicide dose, and g is the dose at inflection point, hence the GR_50_ or LD_50_. IE (Increase effectiveness) = The increase in the effectiveness of Adj [(GR_50_ gly-GR_50_ gly + adj)/(GR_50_ gly)]*100 or [(LD_50_ gly-LD_50_ gly + adj)/(LD_50_ gly)*100].

**Table 2:** Spray retention and contact angle of glyphosate solution for *C. canadensis* and *L. rigidum* populations.

Species	Treatment	Foliar retention *(µL spraying sol. g^-1^dry matter)*^a^	Contact angle (deg)^a^
*C. canadensis*	Glyphosate	350.42b	73.65a
	Glyphosate + Adj	515.60a	65.43b
*L. rigidum*	Glyphosate	307.23b	74.10a
	Glyphosate + Adj	438.60a	67.80b

^a^ Means within a column followed by the same letter are not significantly different at the 5% level as determined by the Tukey test. Mean values ± standard errors of the mean.

**Table 3:** ^14^C-glyphosate absorption and translocation 96 HAT in *C. canadensis* and *L. rigidum* populations.

Species	Treatment	Absorption (%)^a^	Translocation (% ^14^C-absorbed)^b y c^		
			TL	RP	RS
*C. canadensis*	Glyphosate	80.31±3.40b	34.57±1.31a	31.51±2.69a	33.92±3.17a
	Glyphosate + Adj	93.22±1.47a	30.61±2.40b	32.38±1.92a	37.01±2.18a
*L. rigidum*	Glyphosate	78.48±2.39b	37.97±2.15a	29.64±1.83a	32.39±2.68b
	Glyphosate + Adj	90.56±2.45a	28.65±1.82b	32.39±1.17a	38.96±1.75a

^a^ Percentage of ^14^C-glyphosate absorbed from total applied; ^b^ percentage of absorbed; ^c^ TL treated leaf, RP rest of plant, RS root system, (n = 5). Means within a column followed by the same letter are not significantly different at the 5% level as determined by the Tukey test. Mean values ± standard errors of the mean.
